# Determinants of outcome in patients with chronic ischemic left ventricular dysfunction undergone percutaneous coronary interventions

**DOI:** 10.1186/s12872-015-0126-x

**Published:** 2015-10-26

**Authors:** Enrico Ammirati, Valentina Guida, Azeem Latib, Francesco Moroni, Francesco Arioli, Isabella Scotti, Ornella E. Rimoldi, Antonio Colombo, Paolo G. Camici

**Affiliations:** IRCCS Ospedale San Raffaele and Vita-Salute University San Raffaele, Via Olgettina 60, 20132 Milan, Italy; Azienda Ospedaliera Ospedale Niguarda Ca’ Granda, Milan, Italy; A.O. Ospedale di Circolo di Busto Arsizio, Busto Arsizio, VA Italy; Department of Rheumatology, Istituto Ortopedico Gaetano Pini, Milan, Italy; CNR Institute of Clinical Physiology, Milan, Italy; EMO-GVM Centro Cuore Columbus, Milan, Italy; Cardiothoracic Department, San Raffaele Scientific Institute and University, Via Olgettina 60, 20132 Milan,, Italy

**Keywords:** Coronary artery disease, Ischemic systolic left ventricular dysfunction, Heart failure, Coronary revascularization, Percutaneous coronary intervention; stress testing

## Abstract

**Background:**

Percutaneous coronary interventions (PCI) in patients with ischemic systolic left ventricular dysfunction (SLVD) are routinely performed although their impact on prognosis remains unclear.

**Methods:**

We retrospectively evaluated 385 consecutive patients (76 % male, 66 ± 9 years) with SLVD (left ventricular ejection fraction [LVEF] ≤40 %) due to chronic coronary artery disease, who underwent PCI between 1999 and 2009, and explored clinical factors associated with higher risk of death or of a composite of death and hospitalization for acute decompensated heart failure (ADHF).

**Results:**

The median follow-up was 28 months (inter-quartile range 14–46 months). Death and the composite outcome of death and hospitalization for ADHF occurred in 80 (21 %) and 109 (28 %) patients respectively (8.4 and 11.5 per 100 patient-years of follow-up). Insulin-dependent diabetes mellitus (IDDM), multivessel disease, LVEF < 35 %, symptoms of heart failure (HF) emerged both as independent predictors of death (adjusted hazard ratios [HR] 2.64; 1.92, 1.88 and 1.67 respectively) and composite outcome of death and hospitalization for ADHF (adjusted HR 2.22, 1.92, 1.79 and 1.94 respectively). Furthermore advanced age (HR = 1.03) emerged as independent predictors of death and having performed a stress test before PCI correlated with reduced number of deaths and ADHF hospitalizations (HR = 0.60). Of note, PCI significantly reduced the symptom of angina from 63.2 % at baseline to 16.3 % at the last follow up (*p* < 0.0001).

**Conclusions:**

IDDM, symptoms of HF, multivessel disease and LVEF < 35 % appear to be associated with worse outcome patients with ischemic SLVD undergoing PCI, and may be taken into account for optimal risk stratification. On the other hand, performing a stress testing before PCI seems to be associated with a more favorable outcome.

## Background

The estimated prevalence of heart failure (HF) in western countries is of 1–2 % of the general population [1]. Approximately one-half of HF patients are suffering from HF with reduced ejection fraction (HF-REF) [2]. Coronary artery disease (CAD) is the leading cause of systolic left ventricular dysfunction (SLVD), which is the hallmark of HF-REF [3]. Coronary revascularization, by percutaneous coronary intervention (PCI) or coronary artery bypass grafting (CABG), appears to directly target the main pathophysiologic mechanism of ischemic HF-REF. Interestingly, a clearcut survival benefit of revascularization in comparison to medical treatment has never been demonstrated [4–7], Although benefits both on mortality and rate of hospitalization can be achieved with CABG [8, 9], In addition, HF-REF patients with moderate-to-severe systolic dysfunction, i.e. those with the most discouraging prognosis, were largely excluded from currently available trials [7], In the late 70s the Coronary Artery Surgery Study (CASS) first demonstrated that a subgroup of patients with CAD and LV-ejection fraction (LVEF) of 35 to 50 % had a significant survival benefit after CABG compared to medical therapy [10], A more recent prospective randomized trial, the Surgical Treatment for Ischemic Heart Failure (STICH) further addressed the issue of revascularization in patients with severely depressed LVEF. This trial was able to demonstrate the superiority of surgical revascularization versus medical treatment in terms of composite outcome of death and hospitalization for acutely decompensated HF (ADHF) [8]. Whereas the role of PCI in this high-risk, fragile population with SLVD remains to be addressed. The 2014 European Guidelines for revascularization graded CABG as an effective intervention in multivessel CAD patients with LVEF ≤ 35 % as recommendation class I, level of evidence B, whereas PCI was graded as class IIb, evidence level C [11]. Furthermore, few data are available on prognosis and determinants of clinical outcome following PCI in patients with ischemic HF with severe SLVD [12, 13]. A large part of the evidence in this regard derives from studies with limited number of participants [13]. Accordingly, the present analysis including 385 consecutive patients with reduced LVEF ≤40 % (time period 1999–2009) would be among the largest series of patients with ischemic SLVD treated with PCI. We focused on main determinants associated with prognosis in patients with chronic ischemic severe SLVD who had undergone to PCI.

## Methods

### Patient population

From January 1999 to December 2009, 529 consecutive patients with CAD and LVEF ≤40 % underwent myocardial revascularization by PCI at the San Raffaele Hospital in Milan, Italy. Of those, 144 patients underwent urgent PCI for acute coronary syndrome, and were thus excluded from the analysis (30 patients with ST-elevation acute myocardial infarction and 114 with non-ST elevation acute coronary syndromes), leaving 385 subjects for the final analysis. One hundred and forty patients (36 %) underwent stress testing before PCI : single-photon emission computed tomography (SPECT) was used in 61.5 %, stress echocardiography in 20.1 %, stress electrocardiography in 17.1 % and cardiovascular magnetic resonance in 1.4 %. All patients were discharged with aspirin (325 mg/day) and ticlopidine (250 mg BID) or clopidogrel (75 mg daily for 1 to 6 months). Use of β-blockers, statins and ACE inhibitors was left to the discretion of the physician. Clinical follow-up was carried out through visits at the outpatient clinic or by telephone interviews with the patients or their relatives. Ethics: retrospective analysis compliant with Ethical Committee of the Institution.

### Study end-points

The primary end-points of the study were: all-cause mortality and the composite of death and hospitalization for ADHF at a maximum follow up of 48 months. Median follow-up was of 28 months (inter-quartile range 14 to 46 months). We used a hierarchical approach in the time-to-event model, giving priority to death comparing to hospitalization for ADHF in individual patients. We reported 80 deaths (see Table 1), and among non cardiovascular causes of death we had 5 patients who died due to cancer (6.3 %), 2 who had gastrointestinal complications (2.5 %). Nin patients had unknown causes of death (11.2 %) and 6 (7.5 %) patients died as consequences of comorbidity in which ischemic LVSD can be considered a con-cause of death (pneumonia: 2; sepsis; 3, femur fracture: 1).

**Table 1:** Temporal course and causes of deaths in relation to the percutaneous coronary angiography (PCI)

	Within 30 since PCI	Within 1 year since PCI	After 1 year since PCI
Causes of deaths (*n* = 80)			
Sudden death (*n* = 22)	2	10	10
Pulmonary oedema (*n* = 12)	-	4	8
Refractory acute HF (*n* = 10)	-	4	6
Myocardial infarction (*n* = 7)	1	1	5
Pneumonia (*n* = 2)	-	2	-
Ascites (*n* = 1)	-	1	-
Sepsis (*n* = 3)	-	1	2
Abdominal aneurysm rupture (*n* = 1)	-	-	1
Hepatic dysfunction (*n* = 1)	-	-	1
Renal dysfunction (*n* = 2)	-	-	2
Cerebrovascular accident (*n* = 2)	-	-	2
Femur fracture (*n* = 1)	-	-	1
Intestinal occlusion (*n* = 1)	-	-	1
Gastric perforation (*n* = 1)	-	-	1
Cancer (*n* = 5)	-	2	3
Unknown (*n* = 9)	1	1	7

*HF* heart failure

### Statistical analysis

Continuous variables are presented as mean ± standard deviation (SD) or as median (Q1–Q3) when appropriate. Unpaired Student's *t* test and Mann–Whitney *U* test were performed to determine group differences between continuous variables when appropriate. Categorical variables are reported as percentage and are analyzed by Fischer exact test. A multivariable Cox regression model was created with the use of patients’ characteristics in order to identify independent predictors of death and of the composite of death and hospitalization for ADHF. Relative risks were expressed as hazard ratios with associated confidence intervals. Cumulative rate of mortality and of the composite end-point were calculated according to the Kaplan–Meier method. The significance of differences in mortality rates between groups was assessed with the log-rank test. All tests were 2-sided. Probability values <0.05 were considered significant. All analyses were performed using SPSS v17.0 (SPSS Inc., Chicago, Illinois).

## Results

### Characteristics of the patient population

Patients were 66 ± 9 years old and 76 % were male. LVEF was 34 ± 6 %. The majority (74 %) of patients have had at least one previous myocardial infarction. Previous coronary revascularizations by PCI or CABG were performed in 47 and 34 % of the patients, respectively. Most patients had one-vessel disease (70 %). Angina was present in 63 % of the patients whereas symptoms of HF in 22 %. Patients’ characteristics are summarized in Table 2. The PCI procedure significantly reduced the anginal symptom from 63.2 % at baseline (227 out of out of 359 patients; baseline data regarding presence of angina was lacking in 26 [6.7 %] patients) to only in 16.3 % (63 out of 385 patients) at the last follow up (*p* < 0.0001).

**Table 2:** General characteristics of the study population

Baseline Characteristics	Total Population
N°	385
Age - mean (±SD)	66 (±9)
Male sex – *n* (%)	333 (86)
LVEF – mean (±SD)	34 (±6)
Risk factors – *n* (%)	
Family history – *n* (%)	149/357 (42)
Hypertension - *n* (%)	263/366 (72)
Current Smoker – *n* (%)	74/361 (21)
Ex Smoker – *n* (%)	189/361 (52)
Diabetes – *n* (%)	229/385 (59)
IDDM – *n* (%)	52/229 (23)
Hypercholesterolemia – *n* (%)	265/362 (73)
Clinical history – *n* (%)	
Previous MI – *n* (%)	286/384 (74)
Previous CABG – *n* (%)	129/376 (34)
Previous PCI – *n* (%)	174/370 (47)
Symptoms – *n* (%)	
Asymptomatic –*n* (%)	132/359 (37)
Typical and Atypical Angina – *n* (%)	227/359 (63)
N° of diseased vessels – *n* (%)	
One – *n* (%)	254/385 (70)
Diseased Left main stem or LAD	109/254 (43)
Diseased LAD	
Two or more – *n* (%)	125/385 (23)
Symptoms of HF – *n* (%) (yes vs no)	86/385 (22)

Regarding risk factors, clinical history and symptoms we reported the number of patients for whom these data area vailable. *SD* standard deviation, *LVEF* left ventricle ejection fraction, *IDDM* insulin-dependent diabetes mellitus, *MI* myocardial infarction, *CABG* coronary artery bypass grafting, *PCI* percutaneous coronary intervention, and *HF* heart failure

### Determinants of mortality and of composite outcome of death plus hospitalization for ADHF

**Fig. 1:**
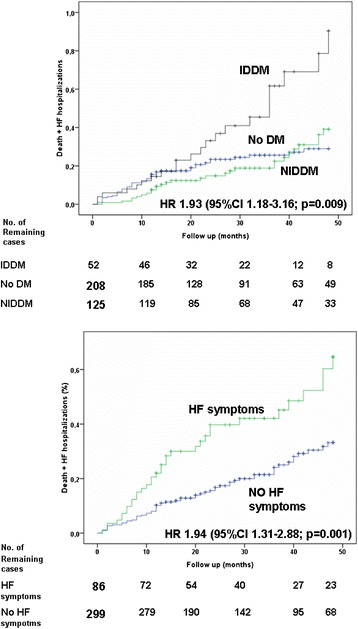
Cumulative rate of the composite end-point (death plus hospitalisation for heart failure -HF-) in patients with insulin-dependent diabetes mellitus (IDDM) versus patients non-insulin-dependent diabetes mellitus (NIDDM) or no diabetes mellitus (NDM) (on the left) calculated according to the Kaplan–Meier method. Similarly cumulative rate of the composite end-point in patients with HF symptoms vs. patients without HF symptoms (on the right). Follow up stops at 28 months

**Fig. 2:**
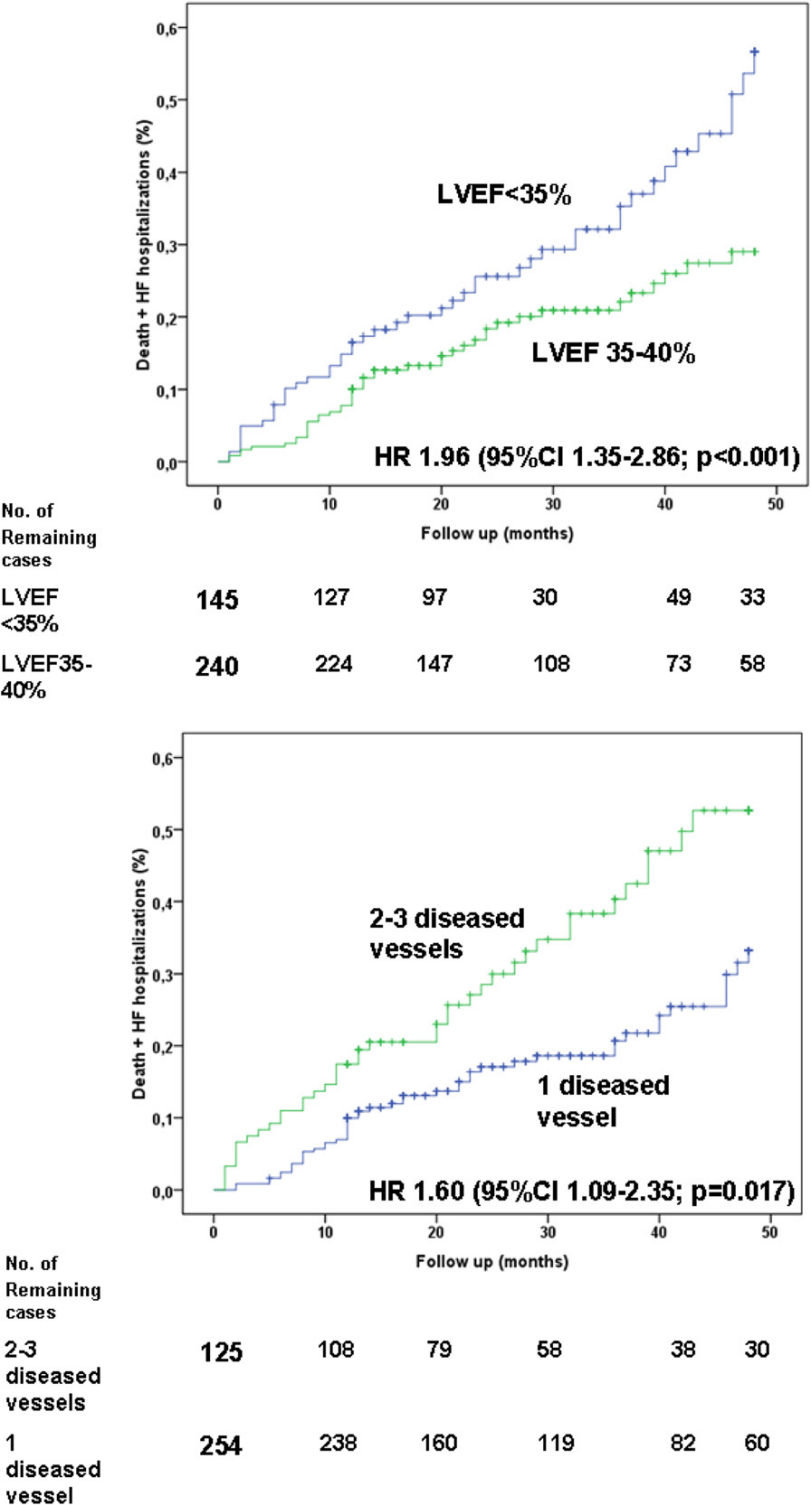
Cumulative rate of the composite end-point (death plus hospitalisation for heart failure -HF-) in patients with left ventricular ejection fraction (LVEF) <35 % vs. patients with LVEF 35–40 % (upper panel) calculated according to the Kaplan–Meier method. Similarly, the lower panel shows cumulative rate of the composite end-point in patients with 2–3 diseased vessels vs. one-diseased vessel (referring to the number of coronary lesions at angiography). Follow up stops at 28 months

**Fig. 3:**
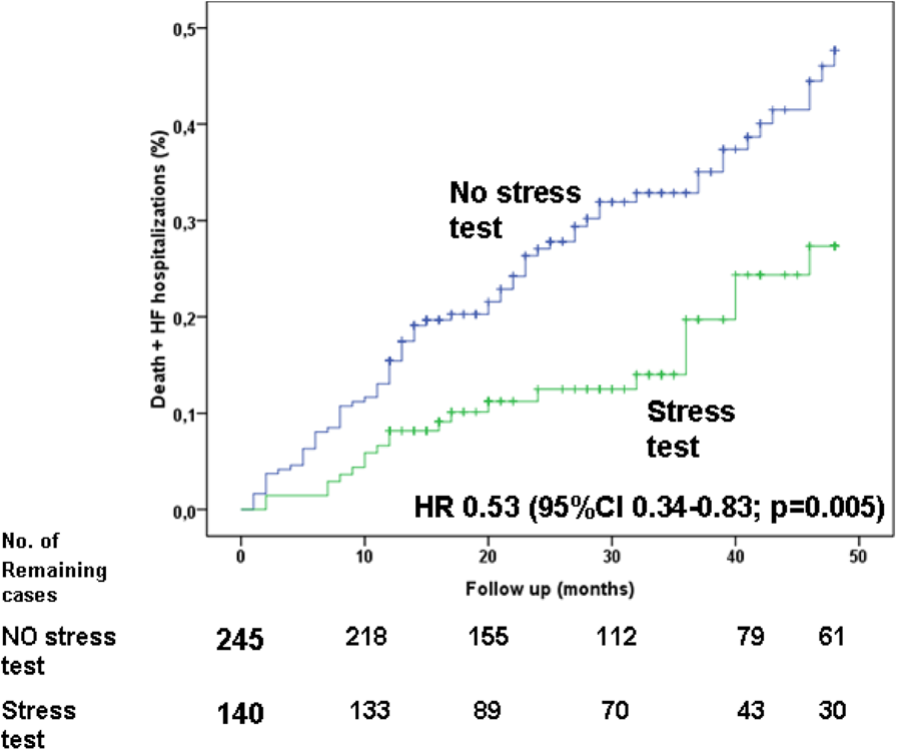
Cumulative rate of composite end-point (death or hospitalization for heart failure -HF-) in patients who did or did not undergo stress testing before PCI. Follow up stops at 28 months

Death and the composite outcome of death and hospitalization for ADHF occurred in 80 (21 %) and 109 (28 %) patients (8.4 and 11.5 per 100 patient-years of follow-up) respectively. As reported in Table 2, univariate analyses showed that insulin-dependent diabetes mellitus (IDDM; hazard ratio [HR] = 2.32), LVEF < 35 % (HR =2.04), multivessel disease (HR =1.63), symptoms of HF (HR = 1.67) and age (HR = 1.03) were significantly associated with death. Similarly, univariate analysis demonstrated that IDDM, symptoms of HF, multivessel disease and LVEF < 35 % were predictors for the composite outcome of death and hospitalization for ADHF. Having performed a stress test before PCI correlated with better outcome (see Table 3 and Figs. 1, 2 and 3).

**Table 3:** Univariate analysis

	Death			Death + ADHF		
	HR	IC 95 %	*P*	HR	IC 95 %	*P*
Age	1.03	1.01–1.06	0.008	1.02	1.00–1.04	0.04
Female sex	0.56	0.26–1.21	0.14	0.85	0,47–1.51	0.57
IDDM	2.32	1.35–4.00	0.002	1.93	1.18–3.16	0.009
HF symptoms	1.65	1.03–2.62	0.036	1.94	1.31–2.88	0.001
Angina symptoms	1.41	0.85–2.34	0.18	1.35	0.87–2.08	0.18
LVEF < 35	2.04	1.32–3.23	0.001	1.96	1.35–2.86	<0.001
Stress testing	0.66	0.40–1.08	0.10	0.53	0.34–0.83	0.005
Multivessel disease	1.63	1.04–2.55	0.032	1.60	1.09–2.35	0.017
Previous MI	0.91	0.55–1.51	0.71	0.89	0.58–1.37	0.59
Previous CABG	1.02	0.63–1.66	0.93	0.96	0.64–1.45	0.85
Previous PCI	0.77	0.49–1.24	0.29	0.69	0.46–1.04	0.074

*ADHF* acute decompensate heart failure, *IDDM* insulin-dependent diabetes mellitus, *HF* heart failure, *LVEF* left ventricle ejection fraction, *MI* myocardial infarction, *CABG* coronary artery bypass graft, *PCI* Percutaneous coronary intervention

Multivariate analysis confirmed that IDDM (HR = 2.64), multivessel disease (1.92), LVEF < 35 % (1.84), symptoms of heart failure (HF; 1.67) and age (1.03) were independent predictors of mortality. Furthermore, IDDM, symptoms of HF, multivessel disease, LVEF < 35 % were confirmed as independent variables associated with the composite outcome of death and hospitalization for ADHF whereas having performed a stress test before PCI was independently associated with better composite outcome. Table 4 summarizes the results of the multivariate analysis.

**Table 4:** Multivariate analysis

	Death			Death + ADHF		
	HR	IC 95 %	*P*	HR	IC 95 %	*P*
Age	1.03	1.00–1.06	0.03	-	-	-
IDDM	2.64	1.50–4.67	0.001	2.22	1.33–3.70	0.002
HF symptoms	1.67	1.02–2.73	0.042	1.94	1.27–2.95	0.002
LVEF < 35	1.84	1.20–2.94	0.006	1.79	1.22–2.63	0.003
Multivessel disease	1.92	1.20–3.07	0.007	1.92	1.28–2.89	0.002
Stress testing	-	-	-	0.60	0.38–0.94	0.025

*ADHF* acute decompensate heart failure, *IDDM* insulin-dependent diabetes mellitus, *HF* heart failure, *LVEF* left ventricle ejection fraction

## Discussion

The present study identified prognostic factors associated with mortality and morbidity outcomes in patients suffering from severe ischemic SLVD undergoing PCI. In this cohort of 385 consecutive patients with moderate-to-severe depression of LVEF, IDDM, symptoms of HF, multivessel disease, LVEF < 35 % and advanced age represented variable associated with poorer outcome. On the other hand, having performed stress testing, mainly using SPECT and stress echocardiography (together these two methodologies account for 81.5 % of all stress tests) before PCI was associated with a better outcome. These data are in accordance with previous studies on coronary revascularization of patients with ischemic SLVD [14]. From our data, it also emerged for the first time that patients who underwent stress testing before PCI have a better outcome. Detailed data on stress testing were not available, so we can only speculate that patients who underwent a stress test could have had better general conditions compared with patients who did not. Alternatively, it can be that information derived by stress testing, e.g. myocardial viability, could have guided the interventional cardiologist to plan better revascularization.

### Predictors of outcome derived from previous studies

In a large Canadian registry, in 1599 patients with CAD and SLVD treated with PCI between 1995 and 2008, Nagendran and colleagues identified age, renal failure, symptoms of HF, diabetes mellitus, peripheral vascular disease, prior myocardial infarction, left main disease, and prior CABG as independent predictors of poor long-term prognosis [14]. Recently, a meta-analysis by Kunadian et al. [13] on outcome of patients with ischemic SLVD treated with PCI identified 19 studies to be considered. Of those, the largest report evaluated 975 patients [15], but only 91 patients had characteristics similar to our cohort. In fact, most of the patients had an LVEF > 45 % and acute coronary syndromes. The Authors identified EuroSCORE as the major determinant of all-cause mortality [15]. Thus, to the best of our knowledge, the present study represents the second largest cohort of patients with ischemic SLVD treated with PCI where analysis was focused on prognostic factors. In a Korean cohort of 329 (age 65 ± 11 years) patients with SLVD treated with PCI in the context of acute coronary syndrome between 2001 and 2006, the independent predictors of mortality at 5-year follow up were LVEF <30 %, serum creatinine >3.0 mg/dL, age older than 65 years, and female gender [16]. Based on our results and previous studies thus we are confident to affirm that advanced age, diabetes mellitus, low LVEF and symptoms of HF are reliably associated with poor outcome in a population quite similar to ours.

### Clinical implications

At present no clinical trial directly compared PCI versus optimal medical therapy in patients with ischemic SLVD [4]. No clinical trial had directly compared PCI with CABG in patients with CAD and SLVD [4]. Data from registries or sub-analyses of large trials seem to suggest that these patients would benefit more from CABG compared with PCI [13, 14, 17–19]. Even if at least 1 registry including patients with LVEF < 50 % where CABG was compared with drug-eluting stent showed similar mortality at 3-year follow up [20]. It is possible that patients with severely depressed LVEF had more frequently complex coronary lesions compared with patients with preserved LVEF and in this setting of patients CABG is superior compared with PCI [21], allowing a more completed revascularization. Among the several potential explanations of a superiority of CABG versus PCI, a more complex coronary lesion Our data show that patients with IDDM, LVEF < 35 % and multivessel disease have a worse outcome following PCI. These patients may be considered for CABG if revascularization is an option. In a recent trial, the Future Revascularization Evaluation in Patients with Diabetes Mellitus: Optimal management of Multivessel Disease (FREEDOM) [22], CABG was associated with reduction of all-cause death in patients with multivessel disease and diabetes compared with PCI. Nevertheless, in this trial less than 3 % of patient had a LVEF <40 %, thus any extrapolation concerning the strategy to adopt in these patients with moderate-severe systolic dysfunction is very limited. Nevertheless, it must be noted a that data from a large registries including PCI using everolimus eluting stent seem to challenge the results of the FREEDOM trial, showing a similar risk of mortality in the long term follow up [23].

A sub-analysis of the STICH trial [24]. Has shown that the presence of a substantial amount of viable myocardium was associated with a greater survival benefit in 601 patients with ischemic SLVD. The same study also reported the counterintuitive finding that the presence of viable myocardium did not identify patients with a differential survival benefit after surgical revascularization, as compared with medical therapy alone. A potential explanation of that finding was that spatial coherence between the region of dysfunctional but viable myocardium and the site of the coronary-artery lesion had not been assessed in that sub-analysis [25]. Finally, the STICH trial results may be affected by selection bias, since stress testing was not mandatory for participation in the trial, and only 399 subjects out of 1212 were analyzed. Furthermore, patients with moderate-to-severe ischemia, which were shown to benefit most from revascularization [26], were under-represented in the STICH sub-analysis [27]. Our data are in line with an interesting sub-analysis of the COURAGE (Clinical Outcomes Utilizing Revascularization and Aggressive Drug Evaluation) trial that observed that the anatomic lesion burden derived from coronary angiography and LVEF were consistent predictors of death and myocardial infarction whereas ischemic burden and treatment assignment (PCI versus medical therapy) were not [28], partially contradicting previous subanalysis from the same study that demonstrated that ischemia reduction had lower unadjusted risk for death or myocardial infarction, particularly if baseline ischemia was moderate to severe [29]. It must be noted that the latter sub-analysis was performed only in patients who had performed SPECT myocardial perfusion imaging at baseline and follow up, thus introducing a potential bias, limiting the analysis to a specific subgroup who performed a test to assess the extent of ischemia.

In our analysis it is of interest that just performing a stress test before PCI was independently associated with a reduction in the cumulative outcome of death and hospitalization for ADHF. Due to the retrospective nature of the analysis it is possible that mostly patients with evidence of ischemia or hibernating myocardium had been subsequently revascularized. This concept could further support the evidence that patients with viable myocardium and SLVD had a better outcome compared with patients with scar independently of the treatment (medical or revascularization) [24].

It must be noted that the sensitivity of non invasive stress testingtends to be higher in patients with single vessel disease compared with multi-vessel disease. This might represent a bias in our study as most of the patients undergoing stress testing had single vessel disease and this might have contributed to the better outcome in the group undergoing stress testing. With regard to the type of stress test that should be used in this type of patients, we believe that SPECT perfusion imaging, which is the test employed in the majority of our patients, is well validated, robust and reproducible technique that provides not only information on the occurrence of reversible perfusion abnormalities following stress, but also on their regional extension. Furthermore, SPECT perfusion imaging provides information on tissue viability and, if cardiac gating is applied, on regional wall motion abnormalities. This is also in line with current guidelines [11], that suggest the use of SPECT perfusion imaging at rest and following stressor stress echocardiography before PCI. It must be also considered the experience of the individual centre and the characteristic of the individual patient (i.e. low thoracic impedance) to determine the selection of the ideal technique.

### Study limitations

This study suffers of potential bias due to its retrospective nature. Several important factors that can affect the outcome of these patients in these analysis are missing, i.e. current HF treatments, data on renal function, presence of ICD, or the results of the stress testing in term of extent of ischemia and hibernated myocardium. However, these data are also missing in other previous published cohorts [15, 30], In this retrospective study data regarding the type of stents (drug eluting stents versus bare metal stents) were available only in 224 patients (58 %), nevertheless among those in which the data were available 80 % received a drug-eluting stent. Since the data derived from a single center, a similar therapeutic approach can be expected in this population.

## Conclusions

PCI seems to be feasible in patients with ischemic LV dysfunction, but evidence of its efficacy and effectiveness are currently lacking. Careful evaluation should be reserved to patients with concomitant IDDM, LVEF < 35 %, HF symptoms, multi-vessel disease and advanced age that represent characteristics associated with a poor prognosis in patients who underwent PCI. A stress testing before PCI can be potentially useful in the selection of patients who can benefit from revascularization. In fact from our analysis undergoing imaging stress testing before the procedure was an independent factor associated with more favourable outcome. Explanations to this finding warrant further investigation.

## Abbreviations

ADHF: Acutely decompensated heart failure
CABG: Coronary artery bypass graft
CAD: Coronary artery disease
CASS: Coronary artery surgery study
COURAGE: Clinical Outcomes Utilizing Revascularization and Aggressive Drug Evaluation
CRT: Cardiac resynchronization therapy
FREEDOM: Future revascularization evaluation in patients with diabetes mellitus: optimal management of multivessel disease
HF-REF: Heart failure with reduced ejection fraction
HR: Hazard ratio
ICD: Implantable cardioverter-defibrillator
IDDM: Insulin dependent diabetes mellitus
LV: Left ventricle
LVEF: Left ventricular ejection function
PCI: Percutaneous coronary intervention
SLVD: Systolic left ventricular dysfunction
STICH: Surgical treatment of ischemic heart failure
